# Commentary: The effect of positive inter-group contact on cooperation: the moderating role of individualism

**DOI:** 10.3389/fpsyg.2024.1452840

**Published:** 2024-09-16

**Authors:** Jiayao Zhu, Da Dong, Xinzhe Jin

**Affiliations:** ^1^Department of Psychology, Shaoxing University, Shaoxing, China; ^2^Center for Brain, Mind and Education, Shaoxing University, Shaoxing, China

**Keywords:** cooperation, individualism, SC-IAT, implicit, cross-strait

Numerous studies have explored cooperative behavior in psychology, yet few have examined this behavior from a cultural perspective within the same group. Culture encompasses the values, norms, thought patterns, behavioral modes, and cultural products shared by a society's members (Song, 2018). It can subtly influence individuals' social cognition (Xie et al., 2008; Yuan et al., 2013) and is also a crucial factor affecting cooperative behavior (Blake et al., 2015; Peysakhovich and Rand, 2016). I was intrigued by a March 2024 article in Frontiers in Psychology that investigated how positive contact and social distance between youth from Chinese Taiwan and mainland China impact cross-strait cooperation under cultural influences. The study also highlighted the mediating role of individualism (Xiao and Li, 2024). Individualism is characterized by a strong belief in self-worth and independence. When faced with the decision to cooperate, individuals with high levels of individualism often prioritize their own interests and benefits, making them less swayed by the depth of their relationship with the partner. As noted by Apanovich et al. (2018) individualism, which prioritizes personal interests, tends to increase the perceived distance between social members, thereby reducing the willingness for positive interaction and cooperation.

In this study, young people in Taiwan were selected as respondents, and the rationale for this choice is both meaningful and far-reaching. Taiwan presents a unique context where both collectivism and individualism coexist. As Taiwan transitions from a traditional agricultural society to a modern one, the influence of modern values on its people has grown increasingly significant (Yang, 1981). Concurrently, Taiwan's youth are becoming more culturally diverse. This study aims to explore and understand how positive contact and social distancing affect the willingness of people on both sides of the Taiwan Strait to cooperate within different cultural and social contexts. Examining Taiwanese youth provides valuable insights into the current status and characteristics of cross-Strait interactions among young people. It also offers new perspectives and methods to enhance exchanges and cooperation between youth on both sides of the Strait. In the context of globalization, fostering exchanges and cooperation among young people is crucial for building mutual understanding and trust. This, in turn, supports the peaceful and stable development of cross-Strait relations. Therefore, this study is of considerable value and significance. It opens a window into the perspectives of young people on both sides of the Taiwan Strait and offers new ideas and directions for promoting the peaceful development of cross-Strait relations.

This study suggests that cooperation is due to the role of social distance, positive contact between groups, and individualism or collectivism. It is undeniable that these factors will affect people's tendency to cooperate in a certain extent. The subjects in this experiment are Taiwan youth. Due to historical and social reasons, Taiwan youth and mainland youth can be regarded as two groups, so there will inevitably be implicit bias between groups. In this study, the author also mentioned that for a long time, under the influence of public opinion, educational institutions and media, some Taiwan youth have formed a certain negative initial impression and prejudice on mainland youth. We believe that in the trust experimental paradigm, individuals may be influenced by their inner bias unconsciously when deciding whether to cooperate or not. Implicit bias is a stable, non-intellectual and not governed by consciousness (Devine and Monteith, 2009). Most people exhibit unconscious, subtle biases that are relatively automatic, dispassionate, indirect, ambiguous, and contradictory. Subtle biases are the basis of ordinary discrimination: being comfortable with one's own in-group, and excluding and avoiding outgroups. This bias stems from the internal conflict between cultural ideals and cultural prejudices (Fiske, 2002). In Taiwan, some people have a negative impression of the mainland, believing that the mainland is an authoritarian regime trying to annex Taiwan. Therefore, in this commentary, I try to put forward the role of “individualism-implicit bias” in the chain mediation, trying to better explain the reasons for the cooperation behavior of Taiwan youth. Please do not misinterpret the intention of this commentary. It is not meant to be a reproach to the authors but rather a contemplation on solutions.

The methodology of this experiment involves a two-step process. First, the initial levels of the subjects are assessed through three different questionnaires to gauge their baseline characteristics. Subsequently, participants engage in an experiment using the trust game paradigm to examine how Taiwanese youth cooperate with their counterparts from mainland China. The trust game is designed with the goal of maximizing monetary gains, which may influence participants' willingness to cooperate in ways that differ from their actual intentions or preferences. While this study focuses on Taiwanese youth, broadening the scope to include a more diverse age range, such as children and the elderly, would enhance the generalizability and representativeness of the findings. Including various age groups could provide a more comprehensive understanding of cooperative behaviors across different life stages, offering richer insights into how age and experience might affect cooperation and interactions between individuals from Taiwan and mainland China.

The implicit bias between Taiwan and the mainland will largely affect cross-strait cooperation. Therefore, based on the study of the original author, I try to put forward the “individualistic implicit bias” as a chain mediating effect on cooperation. First, it is necessary to confirm the existence of implicit bias between Taiwan and the mainland, which can be studied through the Single Category Implicit Association Test (SC-IAT) paradigm (Karpinski and Steinman, 2006). Then, when researchers find the existence of implicit bias, if they want to reduce people's implicit bias, they can imagine intergroup contact, that is, they can mentally imagine the positive interaction between inner and outer group members. This scenario will stimulate the belief of successful contact with outer group members, which is a positive psychological simulation and can increase people's willingness to contact. Reduce the negative emotions of the external group (Stathi et al., 2011). In other words, having a more positive attitude toward the outside group and being more inclined to cooperate with it. Previous studies have found that the length of imaginary time does not affect the effect of imaginary intergroup contact (Turner et al., 2007a). Imagining intergroup contact can effectively reduce prejudice and increase future contact willingness (Husnu and Crisp, 2010). If subjects imagine positive contact with out-group members, the more positive contact they have, the more positive their attitudes toward out-group members will be and the stereotypes will decrease (Turner et al., 2007b). Therefore, in this way, it may be possible to reduce the implicit bias between groups and better promote the willingness of people on both sides of the strait to cooperate.

To more thoroughly investigate the willingness of young people on both sides of the Strait to cooperate, I propose incorporating a variety of social dilemma paradigms in the experimental research methods. For instance, paradigms such as the greatest difference dilemma and the trust dilemma could provide additional insights. Additionally, reducing the decision-making time could be beneficial. Previous studies indicate that under high time pressure, individuals rely more on intuitive decision-making, while reflective processing is more common when there is ample time. This suggests that behaviors under time constraints might reveal more about implicit attitudes (Rand et al., 2012; Yamagishi et al., 2017; Zaki and Mitchell, 2013). For respondent selection, I recommend including a broader age range, as individuals from different age groups may have diverse cultural value orientations that could affect their cooperation tendencies. By comparing cooperative behaviors at different ages, researchers can reveal the inheritance and change of cultural values at different age levels, and provide useful enlightenment for promoting social harmony and progress. Such a broad analysis can help in understanding the generational shifts in cultural values and offer practical guidance for fostering greater cohesion and collaboration within society.
